# Agreement Between Novice Visual Assessment and Classifications Derived from Markerless Motion Capture During Sit-to-Stand Performance in Healthy Adults

**DOI:** 10.3390/healthcare14111549

**Published:** 2026-06-02

**Authors:** Christopher Voltmer, Casey Imperio

**Affiliations:** Doctor of Physical Therapy Program, Touro University, Central Islip, NY 11722, USA; cimperio@touro.edu

**Keywords:** biomechanics, classification, transfers, sit-to-stand, kinematics, motion capture, reliability, clinical assessment, measurement error, inter-rater reliability, ordinal data

## Abstract

**Background:** Visual assessment is commonly used in rehabilitation to evaluate movement quality during functional tasks such as sit-to-stand (STS) transfers. However, the extent to which observational ratings align with classifications derived from portable markerless motion capture systems remains unclear. This study examined agreement between novice observational ratings and motion-capture-derived classifications during STS performance. **Methods:** Fifty healthy adults performed STS transfers across three 18-inch seating conditions (firm, compliant, commode). Two final-year Doctor of Physical Therapy (DPT) students independently rated movement performance using a standardized observational rubric. Simultaneously, a portable markerless motion capture system (Kinotek) recorded joint kinematics, which were converted into ordinal severity classifications to enable a comparison. Inter-rater reliability and agreement were assessed using percent agreement and Krippendorff’s alpha. **Results:** Exact agreement between novice raters was high across all surfaces (82.3–82.9%), while Krippendorff’s alpha values were low despite high exact agreement (α = 0.250–0.323), consistent with restricted scale use. Agreement between observational ratings and motion-capture-derived classifications was low, with negative alpha values across all conditions (α = −0.224 to −0.561), indicating systematic differences in classification patterns. Observational raters more frequently assigned lower severity categories compared to motion-capture-derived classifications. **Conclusions:** Findings demonstrate low chance-corrected agreement under conditions of restricted scale use among novice raters and systematic disagreement between observational and motion-capture-derived classifications during STS performance. These findings reflect differences in classification approaches under the operational definitions used in this study. Motion capture was used as an objective comparator rather than a gold standard, and this study does not establish criterion validity. Further research is needed to evaluate agreement patterns in clinical populations and to examine how different measurement approaches influence functional movement classification.

## 1. Introduction

Functional movement assessment is a central component of rehabilitation practice. Clinicians routinely rely on visual observation to evaluate movement quality, identify compensatory strategies, and inform intervention planning during functional tasks such as sit-to-stand (STS) transfers [1,2]. Despite its widespread use, visual movement assessment is inherently subjective and may be influenced by rater experience, perceptual bias, and task complexity [3,4,5]. Establishing the reliability and measurement properties of observational assessment strategies is therefore essential to support evidence-based clinical decision-making.

Previous investigations examining observational movement assessment have demonstrated variable inter-rater reliability across functional tasks, particularly when movement quality is categorized using ordinal rating scales [6,7]. These findings highlight the importance of understanding how clinician-based ratings correspond with objective biomechanical measurements during dynamic functional activities.

STS performance is one of the most commonly evaluated functional transfers in rehabilitation settings and is frequently used as an indicator of lower extremity strength, balance, and functional mobility across neurologic populations [8,9,10]. Test–retest reliability of STS measures has been demonstrated in individuals with stroke, further supporting its clinical relevance as a functional outcome measure [9]. The task requires coordinated trunk flexion, dynamic weight transfer, and lower extremity force generation; moreover, subtle deviations in movement strategy may not be consistently detected through visual inspection alone.

In clinical environments, STS is performed across a variety of seating conditions, including firm chairs, compliant surfaces, and toileting environments, each of which may influence movement mechanics while maintaining similar seat heights. Portable three-dimensional (3D) motion capture systems have become increasingly accessible in rehabilitation contexts and offer objective kinematic quantification without the infrastructure demands of laboratory-based motion analysis [11,12,13]. Although these systems are widely used in biomechanics research, the degree to which observational movement assessment aligns with portable motion capture classifications during functional transfer tasks remains unclear. To our knowledge, no prior study has examined agreement between visual ordinal classification and portable markerless motion capture during STS performance.

Importantly, this study compares two fundamentally different classification approaches. Observational assessment reflects a global, perceptual evaluation of multi-joint movement patterns, whereas motion-capture-derived classifications are based on joint-level kinematic thresholds. As such, differences between approaches may reflect divergence in underlying constructs rather than disagreement in measurement alone. Framing the comparison in this way is essential for interpreting agreement between perceptual and kinematic classification systems.

Therefore, the primary purpose of this study was to examine inter-rater reliability of visual movement assessment during sit-to-stand performance and to evaluate agreement between observational ratings and motion-capture-derived classifications within the context of differing perceptual and kinematic classification frameworks. Secondary analyses explored patterns of agreement across seating conditions and surface types, and they should be interpreted as exploratory given the fixed-order study design. It was hypothesized that visual raters would demonstrate measurable inter-rater reliability and that agreement between observational ratings and motion-capture-derived classifications would vary across conditions, reflecting differences in classification approaches rather than equivalence in measurement.

## 2. Materials and Methods

### 2.1. Study Design

This cross-sectional study examined (1) inter-rater reliability of observational movement ratings during STS transfers and (2) inter-rater reliability between observational ratings and portable 3D motion capture classifications. Ethical approval was obtained from the Touro University Institutional Review Board (Protocol #20367).

### 2.2. Participants

Fifty healthy adults (26 male, 24 female), aged 21 to 67 (*M* = 27.48, *SD* = 7.07), were recruited from a university community (see Table 1 for full demographics). Inclusion criteria required participants to be ≥18 years of age and able to perform STS transfers independently. Exclusion criteria included balance impairments, recent injury, or inability to provide informed consent. An a priori power analysis was conducted in G*Power 3.1.9.6 to estimate the appropriate sample size for detecting within-subjects effects for the mixed-effects model and was not specifically designed to estimate the precision of agreement metrics. Using an alpha level of 0.05 and 80% power, a sample size of 46 participants was required to detect a small effect (f = 0.15). To account for attrition or potentially unusable data, the target sample was set at 50 participants.

### 2.3. Instrumentation

#### 2.3.1. Visual Assessment

Two novice third-year Doctor of Physical Therapy (DPT) student raters independently scored STS performance using a standardized observational rubric developed for this study, with content validation carried out by three expert clinicians (S-CVI = 0.97) [14,15]. The rubric was designed to capture observable movement deviations across the trunk and lower extremity joints during STS transfers (see Appendix A and Appendix B). Raters evaluated predefined joint regions, including the trunk, hips, knees, and ankles, and identified the presence and severity of observable movement deviations based on a structured scoring framework. Individual joint-level observations were then used to derive a global level-of-support (LOS) severity classification, reflecting the overall magnitude of movement deviation during the task. Within this framework, a noticeable limitation was operationally defined as any observable deviation in joint performance during the transfer, as determined by the rater using the standardized rubric. These binary joint-level determinations (presence or absence of limitation) were then summed to generate a global LOS severity classification according to the predefined scoring thresholds outlined in Appendix B. While the rubric demonstrated strong content validity, additional validation is warranted to further establish its measurement properties.

#### 2.3.2. Motion Capture

A portable 3D motion capture platform (Kinotek) recorded joint range of motion (ROM), asymmetry, and compensatory strategies. The Kinotek is a 3D motion capture platform designed to obtain simultaneous ROM measurements for multiple body parts. The software can measure up to 46 distinct movements and 750 data points per visual analysis [16]. ROM values were categorized into four severity levels (0 = no deficit, 1 = low deficit, 2 = moderate deficit, and 3 = high deficit) based on magnitude outside normative ROM range in either direction. A score of 0 indicated ROM within normative limits. A score of 1 indicated ROM measurements greater than 0° and up to 5° outside of normative range, 2 indicated ROM greater than 5° and up to 10° outside of normative range, and 3 reflected indicated ROM greater than 10° beyond normative range. Continuous ROM data were converted into ordinal categories to enable a comparison with the observational rubric. Normative ROM reference values were derived from system-embedded reference values provided by the Kinotek platform. These values were not adjusted for participant-specific factors such as age, sex, or body size. Although the motion capture system records multiple movement parameters, including asymmetry and compensatory strategies, the ordinal severity classification used in this study was derived solely from ROM deviation thresholds to maintain consistency with the observational rubric.

This transformation was a pragmatic analytic step to facilitate a comparison with the observational rubric and may influence classification patterns. These thresholds were based on deviations from normative ranges but were not independently validated for sit-to-stand severity classification and should be interpreted within this context. The motion capture system was used as an objective comparator rather than a gold standard, and this study does not establish criterion validity. Accordingly, comparisons between observational ratings and motion-capture-derived classifications should be interpreted as differences in classification approaches rather than evidence of accuracy or superiority of either method.

### 2.4. Procedures

Participants performed STS transfers from three 18-inch seating surfaces: (1) firm surface, (2) compliant (soft) surface, and (3) standard-height commode. Surface height was standardized at 18 inches to approximate commonly encountered residential seating conditions while allowing variation in surface compliance and geometry. Inclusion of both firm and compliant surfaces permitted examination of movement performance across variations in surface stability and support characteristics. Prior research has shown that the compliance level of a surface can both alter sensory input and stability [2].

Surface conditions were administered in a fixed sequence (firm → compliant → commode) for all participants. No randomization or counterbalancing was implemented. Each participant performed transfers independently without physical assistance.

During each transfer, both visual raters independently scored movement performance in real time using the standardized observational rubric. Raters were blinded to motion capture output. Simultaneously, the portable 3D motion capture system recorded joint kinematics and movement characteristics.

Although transfers were performed across multiple surfaces, analyses were conducted at the joint level, with each joint-level rating treated as an observational unit. These joint-level observations were used to derive a global LOS classification for each transfer, and repeated observations across joints, surfaces, and raters were accounted for in the analytical approach. Visual ratings and motion-capture-derived classifications were compared within each transfer and surface condition, without pooling across conditions.

### 2.5. Data Analysis

Data were analyzed using JAMOVI software version 2.6.44.0 [17]. First, inter-rater agreement was characterized by calculating percent exact agreement and percent agreement within one rating category (±1), providing initial context for ordinal rating discrepancies beyond reliability coefficients. Due to the ordinal nature of the data, inter-rater reliability and agreement were analyzed using Krippendorff’s alpha, which is a reliability coefficient that quantifies the degree of agreement among raters and accounts for expected chance agreement. In this study, inter-rater reliability refers to agreement between visual raters, whereas agreement refers to comparisons between observational ratings and motion-capture-derived classifications. Unlike other reliability coefficients, Krippendorff’s alpha can be used with nominal, ordinal, interval, and ratio data. Alpha values range from −1 to 1, where 1 indicates perfect agreement, 0 indicates agreement equivalent to chance, and negative values indicate systematic disagreement. When interpreting α, commonly cited thresholds suggest that values <0.67 may be insufficient for drawing reliable conclusions, 0.67 ≤ α < 0.80 may allow for tentative conclusions, and ≥0.80 may be considered reliable [18,19]. However, these thresholds should be interpreted with caution in the presence of imbalanced marginal distributions or restricted category use. To further explore any disagreements elucidated by Krippendorff’s alpha, percentages of disagreement in LOS ratings between novices and the Kinotek were calculated.

Finally, to assess differences between LOS ratings considering within-subject clustering, an ordinal mixed-effects model with LOS as the ordinal outcome, and joint, rater, surface, and the rater × surface interaction as fixed effects was conducted. Participants were included as a random intercept, and the model included 50 participants and 3150 observations.

The primary unit of analysis for agreement calculations was the joint-level rating. For agreement analyses, each joint-level rating was treated as an individual observational unit, with paired ratings compared across raters for the same joint and transfer. Accordingly, agreement coefficients reflect concordance at the joint level rather than the transfer level. For descriptive tables, joint-level observations were summarized within raters, resulting in aggregated counts across participants and surfaces. Each participant contributed repeated observations across joints, surfaces, and raters. For descriptive analyses, joint-level ratings were pooled within each rater, resulting in 1050 observations per rater (50 participants × 3 surfaces × 7 joints). For the mixed-effects model, all joint-level observations across raters were included (N = 3150), with participants included as a random intercept to account for within-subject clustering.

## 3. Results

To quantify patterns of agreement in LOS ratings, both exact agreement and within-1 agreement were calculated between raters. First, the overall distribution of LOS ratings across raters was examined (See Table 2). Descriptive results showed that both novice raters exclusively assigned lower (0–1) LOS ratings, whereas the Kinotek used the full range of the scale, with a greater proportion of higher (3) severity ratings.

Concerning exact agreement, novice raters were more aligned in LOS ratings, with 82.9% (95% CIs [78.5, 86.6]) exact agreement for both the firm and compliant surfaces, and 82.3% (95% CIs [77.8, 85.9]) for the commode. In contrast, novices had consistently lower exact agreement for LOS ratings with the Kinotek device. For the firm surface, agreement was 36.6% (95% CIs [31.7, 41.7]) for Novice 1 vs. Kinotek and 39.7% (95% CIs [34.7, 44.9]) for Novice 2 vs. Kinotek. For the compliant surface, agreement was 21.7% (95% CIs [17.6, 26.3]) and 22.9% (95% CIs [18.7, 27.6]), respectively. Finally, for the commode, agreement was 16.9% (95% CIs [13.3, 21.1]) and 17.4% (95% CIs [13.7, 21.6]), respectively. These findings indicate that while novice raters were giving consistent LOS ratings with each other, their ratings varied from that of the Kinotek. Indeed, within-1 agreement results supported greater agreement between novices, as 100% of LOS ratings across all surfaces were within 1 point for the novice raters. In comparison, between novice raters and the Kinotek, 47% of ratings were within 1 point for the firm surface, 30% for the compliant surface, and approximately 24% for the commode. This pattern of results indicates that while novices were in agreement, the discrepancies between the novices and the Kinotek are systematic and vary with surface type. Confusion matrices between each novice rater and the Kinotek device were created to further characterize discrepancies in LOS ratings (See Table 3 and Table 4). As previously shown in Table 2, novices exclusively assigned LOS ratings of 0 and 1, whereas the Kinotek used the full range of the scale. The discrepancies between novices and the Kinotek were most pronounced when the Kinotek assigned moderate or high LOS ratings (2 and 3), which were frequently classified as no to little severity by the novices (0 and 1).

Due to the ordinal nature of the data, Krippendorff’s alpha was used to calculate inter-rater reliability across surfaces between visual raters and between visual raters and motion capture. Alpha coefficients for each rater comparison are presented in Table 5.

Novice raters demonstrated low Krippendorff’s alpha values across surfaces (α = 0.250–0.323) despite high exact agreement. This apparent discrepancy likely reflects restricted use of ordinal categories and imbalanced marginal distributions, which are known to deflate chance-corrected agreement coefficients. In the present study, both novice raters used only the lowest categories (0–1), resulting in severe range restriction. Therefore, alpha values in this context should be interpreted with caution and in conjunction with descriptive agreement metrics. Agreement between novices and the motion capture system was consistently negative across all transfer surfaces (α = −0.224 to −0.561), indicating systematic differences in classification patterns rather than random disagreement.

**Figure 1:**
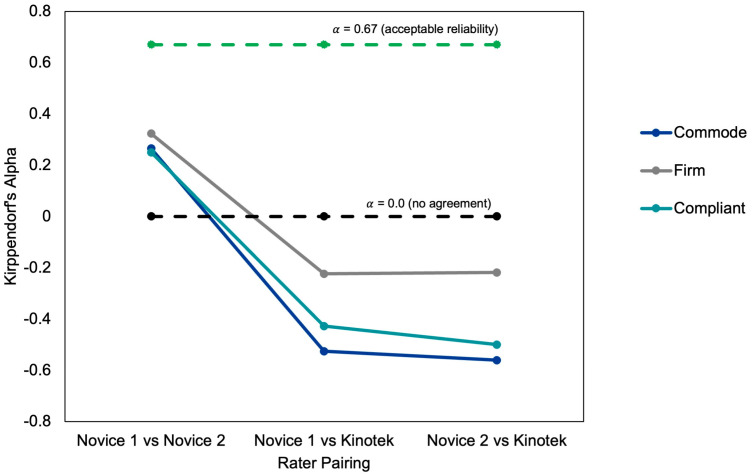
Inter-rater reliability, quantified using Krippendorff’s α, is displayed for each assessment pairing across commode, firm-, and compliant-surface STS transfers. Positive α values indicate agreement greater than chance, whereas negative values indicate systematic disagreement. The green dashed line represents a commonly accepted threshold for acceptable reliability (α = 0.67), and the black dashed line represents no agreement (α = 0). Agreement between novice raters was low across all surfaces (95% CIs: commode [0.10, 0.43], firm [0.17, 0.47], compliant [0.08, 0.41]). In contrast, comparisons between novice raters and the Kinotek device were consistently negative across surfaces (95% CIs: Novice 1 vs. Kinotek commode [−0.63, −0.42], firm [−0.35, −0.10], compliant [−0.54, −0.32]; Novice 2 vs. Kinotek commode [−0.67, −0.46], firm [−0.34, −0.09], compliant [−0.61, −0.39]), indicating systematic differences in classification patterns between observational and motion-capture-based approaches.

This pattern was observed consistently across all surfaces when comparing both novices to Kinotek, respectively (see Figure 1). However, this pattern may reflect differences in measurement approaches, as continuous ROM outputs from the Kinotek were transformed into ordinal LOS categories to enable a comparison with novice ratings.

Confidence intervals for novice vs. Kinotek comparisons were entirely below zero across all surfaces, indicating systematic disagreement in classification patterns (See Table 5). In contrast, confidence intervals for novice vs. novice comparisons were all above zero but below accepted reliability thresholds, indicating low but consistent agreement in classification patterns.

Cohen’s kappa (quadratic weights) was also conducted to further assess inter-rater agreement while accounting for the ordinal nature of the LOS scale. Agreement between novice raters was fair (κw = 0.28, 95% CI [0.21, 0.36], *p* < 0.001), which indicated consistent scoring patterns for the visual raters. Agreement between the novice raters and the Kinotek was negligible (Novice 1 vs. Kinotek: κw = 0.01, 95% CI [−0.00, 0.02], *p* = 0.169; Novice 2 vs. Kinotek: κw = 0.01, 95% CI [−0.00, 0.02], *p* = 0.219). The results from the Cohen’s kappa analyses are consistent with the results of Krippendorf’s alpha, showing that though novice raters showed internal consistency, the visual raters’ classifications systematically differed from motion-capture-derived ratings.

**Figure 2:**
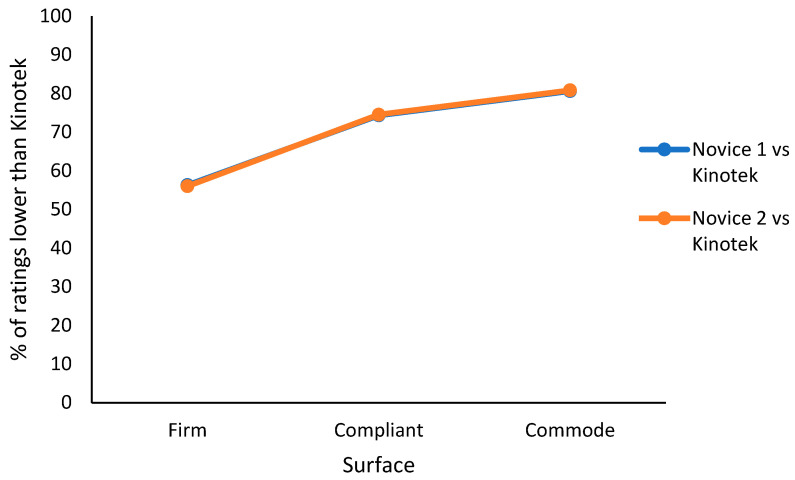
Direction of disagreement between each novice rater and the Kinotek device across surfaces. Data represent the percentage of trials that novices assigned lower LOS ratings than the Kinotek. Percentages were calculated as the proportion of negative difference scores (novice − Kinotek). As surface difficulty increased, the proportion of ratings assigned by novice raters that were lower than those of the Kinotek also increased. Both novices showed similar proportions of lower LOS ratings relative to the Kinotek across all surfaces.

Next, direction of disagreement between the Kinotek and the novices was calculated to quantify the discrepancies in LOS ratings. Novices more frequently gave lower LOS ratings compared with the Kinotek, and 56% of their ratings were lower than the Kinotek ratings for the firm surface, 74% were lower for the compliant surface, and 81% were lower for the commode (see Figure 2). These patterns of disagreement suggest that novice raters more frequently assigned lower LOS classifications relative to motion-capture-derived classifications during STS transfers, particularly in the compliant and commode conditions; however, these differences should be interpreted in the context of the fixed-order design and cannot be attributed solely to surface characteristics. Novices most frequently assigned low severity ratings, whereas motion capture analysis identified a greater frequency of moderate and high-severity movement deficits across all surfaces. These differences may reflect variation in how movement characteristics are identified and classified across approaches during STS transfers performed on more challenging surfaces, such as the commode.

Finally, an ordinal mixed-effects model with a proportional odds cumulative link (logit) was conducted to examine differences among raters, surfaces, and joints while accounting for within-subjects clustering. The assumption of proportional odds was evaluated by examining the consistency of parameter estimates across cumulative logits and by assessing overall model fit; no substantial deviations were observed. In addition, model fit indices (AIC = 4058.62, BIC = 4167.61, log-likelihood = −2011.31) and the likelihood ratio (χ^2^(14) = 1816.57, *p* < 0.001) indicated acceptable fit compared to an intercept-only model. The variance of the random intercept was 0.295 (SD = 0.543; ICC = 0.082), indicating modest within subject clustering. The model showed that LOS ratings differed across all predictors, including joint (χ^2^(6) = 201.816, *p* < 0.001), rater (χ^2^(2) = 1140.682, *p* < 0.001), and surface (χ^2^(2) = 10.666, *p* = 0.005). The interaction between rater and surface was also significant (χ^2^(4) = 42.968, *p* < 0.001), indicating that discrepancies between raters differ by surface. Parameter estimates indicated that the Kinotek device had substantially greater odds of assigning higher LOS ratings compared to the reference novice rater (OR = 38.98, 95% CI [30.44, 49.93], *p* < 0.001). Furthermore, lower odds of higher LOS ratings were observed on firm surfaces compared to the commode condition (OR = 0.68, 95% CI [0.54, 0.86], *p* = 0.001). Follow-up examination of the interaction via parameter estimates showed that differences in LOS ratings between novice raters and the Kinotek was greatest for the commode condition, specifically for one novice rater (*B* = −1.305, *p* < 0.001; OR = 0.27, 95% CI [0.16, 0.46]), which indicates a substantially lower odds of assigning higher LOS ratings relative to the Kinotek under this condition. All other rater × surface interaction terms were not significant (all *p*’s > 0.05). Full model parameter estimates are provided in Supplementary Table S1.

## 4. Discussion

This study examined inter-rater reliability of observational movement assessment during STS transfers and evaluated agreement between observational ratings and portable 3D motion capture classifications across three commonly encountered 18-inch seating conditions. The primary findings were as follows: (1) low Krippendorff’s alpha values despite consistent exact agreement between novice raters, (2) systematic disagreement between novices and motion capture ratings, and (3) motion capture classifications reflecting a higher frequency of moderate-to-high deviation ratings relative to visual scoring.

The discrepancy between low Krippendorff’s alpha values and high exact agreement likely reflects a restricted use of the rating scale and limited discrimination across the ordinal LOS categories. Range restriction is a phenomenon often seen in research, in which the rater does not make full use of the scale, and can greatly impact IRR and lead to underestimated reliability coefficients [18]. In this case, raters may have limited use of higher severity categories, restricting the range of ratings used. Additionally, raters may have demonstrated limited discrimination among the ordinal scale rating categories, potentially assigning similar LOS ratings to biomechanical deficits that may have warranted higher severity classifications. Observational scoring of dynamic functional tasks requires rapid perceptual integration of multi-joint movement patterns and interpretation of ordinal criteria, processes known to be influenced by rater experience and attentional focus [4,7,19]. Previous work examining observational assessment in functional mobility tasks has similarly demonstrated variability in rater agreement, particularly when subtle biomechanical deviations are present [3,4,5]. The present findings extend this literature by demonstrating low chance-corrected agreement under conditions of restricted scale use even when surface height is standardized. This interpretation was also supported by the weighted kappa analyses, which also demonstrated fair agreement between the novice raters, but negligible agreement between both novice raters and the Kinotek classifications.

The negative Krippendorff’s alpha values observed between visual raters and motion capture classifications indicate systematic disagreement beyond chance [20,21], suggesting directional divergence in classification patterns rather than random variability. This pattern suggests that novice raters may have more frequently assigned lower severity classifications relative to motion-capture-derived classifications.

An important consideration when interpreting these findings is the potential mismatch in the constructs being measured by the two approaches. This reflects a fundamental difference between a global perceptual construct and joint-level kinematic thresholding, which may capture distinct aspects of movement performance. The observational rubric reflects a global assessment of visible movement deviations across multiple joints during STS performance, whereas the motion-capture-derived classifications are based on joint-level kinematic deviations from normative ranges. These approaches capture different aspects of movement impairment, including perceptual integration of multi-joint coordination versus quantification of discrete joint excursions. As a result, disagreement between methods may reflect differences in how movement deficits are defined and operationalized rather than inconsistency or inaccuracy of either approach.

In this study, motion capture-derived classifications more frequently fell within moderate and high deviation categories relative to the predominantly low severity ratings assigned by novice raters, reflecting differences in classification approaches under the operational definitions used in this study. Such patterns may reflect differences in measurement resolution between objective kinematic quantification and visually interpreted ordinal thresholds. Sensor-based systems quantify joint excursions, asymmetry, and movement timing with high temporal precision [22,23,24,25,26], which may reflect differences in how movement characteristics are quantified relative to observational scoring.

The inclusion of three surface conditions (firm, compliant, commode) allowed for an evaluation of agreement across varied but height-controlled environmental contexts. Surface compliance and geometry are known to influence trunk strategy and lower extremity loading during STS performance [8,9,10]. Disagreement patterns were observed consistently across surfaces, suggesting that variability in classification was not confined to a single environmental condition. Indeed, novice raters diverged from the Kinotek more in the compliant and commode conditions, with the greatest discrepancy observed in the commode condition. This pattern may reflect differences in classification under changing task conditions; however, given the fixed-order design, these findings may also reflect cumulative effects of practice, fatigue, or task exposure. Compliant and commode surfaces may introduce variability in movement strategy, postural control, and load distribution. Nonetheless, these effects cannot be isolated from potential order-related influences in the present design [12,27]. Importantly, because surface conditions were administered in a fixed sequence, potential order or fatigue effects cannot be excluded and should be considered when interpreting findings. Accordingly, surface-related differences should be interpreted cautiously, as condition and order effects cannot be disentangled in the current design.

This pattern may reflect inherent limitations in the real-time visual assessment of complex, multi-joint movement tasks. Sit-to-stand performance requires rapid integration of trunk, hip, knee, and ankle mechanics, and subtle deviations in timing, asymmetry, or joint excursion may not be readily perceptible without objective quantification. Because raters performed assessments in real time without video replay, cognitive demands and observational constraints may have influenced scoring consistency. As task complexity increases, particularly across varying surface conditions, these demands may further reduce the ability to consistently identify and grade movement deviations. This reflects typical clinical and educational settings, in which novice clinicians often rely on real-time observation without the benefit of replay, and it highlights the potential value of objective measurement tools as supportive references during the development of movement assessment skills.

These findings have implications for the interpretation of observational movement assessment rather than direct clinical decision-making. Visual assessment of functional movement is widely used in practice; however, the low Krippendorff’s alpha values and systematic disagreement observed in this study suggest variability in how movement deficits may be identified and classified under the conditions examined. These findings highlight considerations in how different movement assessment approaches are interpreted and underscore the need for further research evaluating agreement in clinical populations and among experienced clinicians. In this context, objective measurement approaches may serve as complementary tools to support our understanding of movement classification rather than being direct determinants of clinical decisions.

The findings also have implications for physical therapy education. The use of novice raters in this study reflects early professional training and highlights variability in the development of observational movement analysis skills. These results suggest that structured training approaches, along with the incorporation of objective feedback tools such as portable motion capture systems, may enhance the development of clinical reasoning and support development of consistency in movement assessment among developing clinicians. It is important to note that these findings should be interpreted within the context of novice raters and may reflect early-stage development of observational assessment skills rather than inherent limitations of visual assessment.

These findings highlight classification differences between observational and kinematic-based assessment approaches during functional transfer tasks. Portable motion capture systems have demonstrated reliability and validity in gait and postural control analysis [12,27,28], and advances in portable markerless motion capture technology offer a feasible approach for integrating objective movement analysis into both clinical and educational environments. Importantly, these differences should not be interpreted as evidence of superiority, but rather as reflecting differences in classification frameworks. These systems may serve as complementary tools to traditional observational assessment by providing quantifiable data that can support standardized evaluation and inform future research on movement classification.

## 5. Limitations

Several limitations should be considered when interpreting these findings. First, participants were healthy adults recruited from a university community. The use of a healthy cohort allowed for a controlled evaluation of agreement and classification patterns across standardized conditions, without the added variability introduced by clinical pathology. This approach is also consistent with educational practice, in which foundational assessment skills are developed in controlled environments before being applied to more complex clinical populations. Findings may not generalize to clinical populations in which movement deviations are more pronounced or heterogeneous. Agreement patterns may differ in individuals with neurologic, musculoskeletal, or balance impairments.

Second, surface conditions were administered in a fixed sequence for all participants. The absence of randomization or counterbalancing introduces the possibility of order effects, including practice, fatigue, or adaptation across successive transfers. Although surface height was standardized at 18 inches, differences in surface compliance and commode geometry may have influenced trunk strategy, joint loading, and movement variability. Systematic bias therefore cannot be ruled out, and surface-related findings should be interpreted with caution.

Third, continuous motion capture data were converted to ordinal classifications to align with the observational rubric and permit direct agreement analysis. While this approach enabled a comparison between modalities, categorization may have reduced granularity of continuous kinematic information and influenced agreement coefficients. This transformation of continuous ROM data into ordinal categories may introduce classification bias and influence agreement metrics. Previous studies have evaluated motion capture systems against established reference standards to assess validity in controlled contexts [29]; however, such validation was beyond the scope of the present study, which focused on agreement between classification approaches rather than criterion validity.

Fourth, visual raters were novice DPT students. Results may not reflect the reliability or assessment patterns of experienced clinicians. However, the inclusion of novice raters provides an insight into observational consistency during early professional training.

Finally, the a priori power analysis was based on detecting within-subject effects for the mixed-effects model and was not specifically aligned with the primary aims of evaluating inter-rater reliability. Sample size determination for agreement studies is more appropriate based on the expected precision of the agreement coefficients (e.g., Krippendorff’s α or weighted kappa). Thus, the precision of agreement estimates was instead characterized using 95% confidence intervals for reliability and agreement metrics.

## 6. Future Directions

The current study evaluated agreement between classification systems but did not assess predictive validity or clinical outcomes. Future research should examine agreement patterns in clinical populations (including individuals with neurologic or musculoskeletal impairments, where movement deviations may be more pronounced) and determine whether integration of objective movement feedback improves rater consistency or clinical decision-making over time. Further, establishing clinically meaningful thresholds of disagreement and understanding how differences in classification approaches influence clinical decision-making remain important areas for further investigation. Additionally, longitudinal studies evaluating whether objective feedback improves observational rater consistency over time would further clarify the role of technology-assisted movement assessment in rehabilitation education and practice.

## 7. Conclusions

This study demonstrated low Krippendorff’s alpha values despite high exact agreement among novice raters of observational movement assessment during STS transfers and systematic disagreement between visual ratings and portable 3D motion capture classifications across three seating conditions. Motion capture classifications reflected a higher frequency of moderate-to-high movement deviation ratings relative to visual scoring, consistent with differences in classification patterns between assessment approaches in this healthy cohort. These findings highlight limitations in observational consistency among novice raters and support the continued investigation of objective motion analysis tools as complementary approaches for movement assessment in rehabilitation practice and education.

## Figures and Tables

**Table 1:** Demographic information.

Category		Frequency
Sex	Male	26
	Female	24
Race	Asian	19
	Black/African American	1
	White	27
	Prefer not to answer	3
Ethnicities	Hispanic/Latino	5
	Not Hispanic/Latino	36
	Prefer not to answer	9
School year	1st	19
	3rd	11
	Post Grad	20
Pre-existing conditions	Yes	2 *
	No	48

* One participant had history of herniated disks, and another had myofascial pain syndrome.

**Table 2:** Frequency distribution of severity ratings (pooled across surface).

LOS	Novice 1	Novice 2	Kinotek
0	875 (83.3%)	931 (88.7%)	306 (29.1%)
1	175 (16.7%)	119 (11.3%)	41 (3.9%)
2	0 (0.0%)	0 (0.0%)	93 (8.9%)
3	0 (0.0%)	0 (0.0%)	610 (58.1%)
Total	1050	1050	1050

Note. LOS = level of severity. Values represent frequencies with column percentages in parentheses. Data are pooled across STS surfaces.

**Table 3:** Confusion matrix comparing Novice 1 and the Kinotek device.

	Kinotek LOS				
Rater 1 LOS	0	1	2	3	Total
0	258 (29.5%)	36 (4.1%)	87 (9.9%)	494 (56.5%)	875
1	48 (27.4%)	5 (2.9%)	6 (3.4%)	116 (66.3%)	175
Total	306 (29.1%)	41 (3.9%)	93 (8.9%)	610 (58.1%)	1050

Note. LOS = level of severity. Values represent frequency counts with row percentages in parentheses. Row percentages represent the proportion of ratings within each Rater 1 severity level. Data are pooled across STS surfaces.

**Table 4:** Confusion matrix comparing Novice 2 and the Kinotek device.

	Kinotek LOS				
Rater 2 LOS	0	1	2	3	Total
0	276 (29.6%)	37 (4.0%)	84 (9.0%)	534 (57.4%)	931
1	30 (25.2%)	4 (3.4%)	9 (7.6%)	76 (63.9%)	119
Total	306 (29.1%)	41 (3.9%)	93 (8.9%)	610 (58.1%)	1050

Note. LOS = level of severity. Values represent frequency counts with row percentages in parentheses. Row percentages represent the proportion of ratings within each Rater 2 severity level. Data are pooled across STS surfaces.

**Table 5:** Inter-rater reliability (Krippendorff’s alpha) for all raters and transfer surfaces.

	Commode	Firm	Compliant
All Raters	−0.242	−0.059	−0.208
1 vs. 2	0.265 [0.10, 0.43]	0.323 [0.17, 0.47]	0.250 [0.08, 0.41]
1 vs. Kinotek	−0.526 [−0.63, −0.42]	−0.224 [−0.35, −0.10]	−0.428 [−0.54, −0.32]
2 vs. Kinotek	−0.561 [−0.67, −0.46]	−0.219 [−0.34, −0.09]	−0.500 [−0.61, −0.39]

Rater 1 = novice, Rater 2 = novice. Values represent Krippendorff’s α with 95% bootstrap confidence intervals in brackets. Krippendorff’s alpha was negative for all ratings between Kinotek and both student raters, indicating disagreement among raters on LOS of deficits during transfers. In contrast, alpha levels were positive between student raters, indicating agreement. However, in this case, alpha values were low despite high exact agreement, which may reflect restricted use of ordinal categories and imbalanced rating distributions.

## Data Availability

The dataset presented in this study is openly available at Figshare at [10.6084/m9.figshare.32077557]. The dataset includes de-identified, derived data and an accompanying codebook to support reproducibility.

## Supplementary Materials

The following supporting information can be downloaded at: https://www.mdpi.com/article/10.3390/healthcare14111549/s1, Table S1: Parameter estimates from the ordinal mixed-effects (proportional odds) model.

## Appendix A

**Dear experts,**

This rating scale contains three domains related to assessing biomechanical issues in sit-to-stand transfers. We request your expert judgment on the degree of relevance of each item to the measured domains. You should base your review on the definitions for each domain provided below. We request that you be as objective and constructive as possible when reviewing this scoring form. Please use the scale below:

**Degree of relevance:**

1 = there is no relevance to the measured domain

2 = there is some relevance to the measured domain

3 = there is sufficient relevance to the measured domain

4 = there is excellent relevance to the measured domain

**Please look at the scoring form below and rate the degree of relevance for the following domains:**

1. Are these appropriate joints that you would look at for sit-to-stand transfer?
2. Is utilizing a binary yes/no scale appropriate for confirming if there is a biomechanical deficit during sit-to-stand transfer?
3. Is utilizing a rating scale of low (1), medium (2), and high (3) appropriate for measuring level of severity of biomechanical issues in each joint during a sit-to-stand transfer?

## Appendix B

**Participant ID:____________**

**Rater ID:___________**

**Transfer (Please circle 1):** Soft      Firm      Commode

During each phase, each relevant individual LE joint motion will be assessed and assigned a numerical value of 1 or 0 depending on if they function with or without limitations.

**Biomechanical Deficit Ratings:**

1 = Joint functions with noticeable limitations

0 = Joint functions without noticeable limitations

**Level of severity Ratings**

0 = none

1 = low

2 = medium

3 = high

**Transfer Assessed**

**Joint**	**Biomechanical Deficit**	**Level of Severity**
HipLeft		
HipRight		
Trunk/Spine		
KneeLeft		
KneeRight		
AnkleLeft		
AnkleRight		

**How to score each transfer:** Add up the ratings from each phase. Use the scale below to give a global rating for each phase:

3 = 6 or more biomechanical issues

2 = 3 to 5 biomechanical issues

1 = 2 or less biomechanical issues

0 = No biomechanical issues

Higher scores indicate greater observed movement deviation during the transfer.
