# Supplementary material for: Agreement Between Novice Visual Assessment and Classifications Derived from Markerless Motion Capture During Sit-to-Stand Performance in Healthy Adults
Source: Healthcare (Basel). 2026 Jun 2;14(11):1549. doi: 10.3390/healthcare14111549 (PMC13257056; doi:10.3390/healthcare14111549)
Supplement: Supplementary file 1 [file healthcare-14-01549-s001.zip › healthcare-4253459-supplementary.pdf]

Supplementary Table S1. Parameter estimates from the ordinal mixed-effects (proportional odds) model

Predictor	Contrast	B	SE	OR	95% CI Lower	95% CI Upper	z	p
**Intercept (Threshold)**	**0|1**	**0.926**	**0.094**	**2.524**	**2.098**	**3.038**	**9.807**	**<.001**
**Intercept (Threshold)**	**1|2**	**1.994**	**0.103**	**7.347**	**6.001**	**8.995**	**19.316**	**<.001**
**Intercept (Threshold)**	**2|3**	**2.415**	**0.108**	**11.185**	**9.049**	**13.826**	**22.327**	**<.001**
Joint	AnkleRight_LOS - AnkleLeft_LOS	0.055	0.177	1.056	0.746	1.495	0.307	0.758
**Joint**	**HipLeft_LOS - AnkleLeft_LOS**	**1.242**	**0.174**	**3.462**	**2.463**	**4.866**	**7.147**	**<.001**
**Joint**	**HipRight_LOS - AnkleLeft_LOS**	**1.339**	**0.173**	**3.815**	**2.718**	**5.356**	**7.738**	**<.001**
**Joint**	**KneeLeft_LOS - AnkleLeft_LOS**	**1.049**	**0.176**	**2.856**	**2.022**	**4.034**	**5.958**	**<.001**
**Joint**	**KneeRight_LOS - AnkleLeft_LOS**	**1.059**	**0.176**	**2.883**	**2.041**	**4.073**	**6.008**	**<.001**
**Joint**	**Trunk/Spine_LOS - AnkleLeft_LOS**	**-0.916**	**0.204**	**0.400**	**0.268**	**0.597**	**-4.494**	**<.001**
**Rater**	**Novice 2 vs Novice 1**	**-0.426**	**0.132**	**0.653**	**0.504**	**0.846**	**-3.229**	**0.001**
**Rater**	**Kinotek vs Novice 1**	**3.663**	**0.126**	**38.982**	**30.437**	**49.926**	**29.014**	**<.001**
**Surface**	**Firm - Commode**	**-0.383**	**0.118**	**0.682**	**0.540**	**0.860**	**-3.235**	**0.001**
Surface	Compliant - Commode	-0.150	0.123	0.861	0.677	1.094	-1.225	0.221
Rater x Surface	Novice 2 x Firm (vs Novice 1 x Commode)	0.072	0.316	1.075	0.578	1.997	0.229	0.819
**Rater x Surface**	**Kinotek x Firm (vs Novice 1 x Commode)**	**-1.305**	**0.265**	**0.271**	**0.161**	**0.456**	**-4.926**	**<.001**
Rater x Surface	Novice 2 x Compliant (vs Novice 1 x Commode)	-0.285	0.329	0.752	0.395	1.433	-0.866	0.387
Rater x Surface	Kinotek x Compliant (vs Novice 1 x Commode)	-0.289	0.266	0.749	0.444	1.261	-1.087	0.277

Note. B = log-odds coefficient; OR = odds ratio; CI = confidence interval. Reference categories were AnkleLeft_LOS (joint), novice rater 1, and commode surface. Threshold parameters represent cumulative logit cut points. Significant effects (p < .05) are shown in bold.
